# Nutritional resources of the yeast symbiont cultivated by the lizard beetle *Doubledaya bucculenta* in bamboos

**DOI:** 10.1038/s41598-021-98733-y

**Published:** 2021-09-28

**Authors:** Wataru Toki, Dan Aoki

**Affiliations:** Graduate School of Bioagricultural Sciences, Nagoya University, Furo-cho, Chikusa-ku, Nagoya, Aichi 464-8601 Japan

**Keywords:** Microbial ecology, Entomology, Fungal ecology

## Abstract

Symbiotic fungi of wood-inhabiting insects are often considered to aid wood digestion of host insects when the associated fungi can assimilate wood-associated indigestible materials. In most cases, however, the components of wood that are utilized by fungal symbionts remain poorly understood. The lizard beetle *Doubledaya bucculenta* (Coleoptera, Erotylidae, Languriinae) farms the symbiotic yeast *Wickerhamomyces anomalus* inside the cavity of host bamboo internodes, which serves as food for larvae. To determine the carbon sources of the internodes serving as nutritional substrates for *W. anomalus*, we used ion exchange chromatography measurements to analyze free and structural sugar compositions in fresh pith (FP), yeast-cultured pith (YP), and larva-reared pith (LP) of internodes. Glucose and fructose were the major free sugars in FP and markedly decreased in YP and LP. For structural sugars, no sugar significantly decreased in YP or LP compared with FP. Carbon assimilation tests showed that *W. anomalus* assimilated glucose, mannose, fructose, and sucrose strongly, xylose and cellobiose moderately, and xylan weakly. Elemental analysis revealed that the compositions of carbon, hydrogen, and nitrogen were not significantly different among tissue types. These results suggest that *W. anomalus* does not consume bamboo-associated indigestible sugars but most free sugars, mainly glucose and fructose, in the pith. Our findings suggest that a symbiont’s abilities may not always benefit its host in nature.

## Introduction

In fungus-farming mutualism practiced by insects, a host insect provides its fungal symbiont with a nutritional substrate. For example, in leaf cutter ant-fungus systems, the ants cut plant tissue and transport it into their breeding system, where they inoculate the harvested plant material with a mutualistic fungus^1^. In ambrosia beetle-fungus systems, beetles are well-known to excavate tunnel systems within the sapwood of trees, where they inoculate the woody tissue (e.g., gallery walls) with their mutualistic fungus^2^. Thus, the woody tissue serves as the main nutritional substrate for the ambrosia beetle fungi. This is also the case for some other mutualistic associations between wood-inhabiting insects and fungi, including ship timber beetles (Lymexylidae) and woodwasps (Hymenoptera)^2,3^.

Wood is low in nitrogen and mainly consists of indigestible polymers such as cellulose, hemicelluloses, and lignin, and thus, it is difficult for insects to digest^4^. However, fungal cultivars of some ambrosia beetle lineages can decompose wood-associated polysaccharides such as cellulose and hemicelluloses including xylan^5,6^. Ship timber beetles bore tunnels into wood and farm fungal symbionts within the tunnels for food^7^. Fungi isolated from the ship timber beetle *Elateroides flabellicornis* are capable of assimilating wood-associated sugars including xylose, arabinose, and xylan^8^. Symbiotic microbes of wood-inhabiting insects such as bacteria, yeasts, and other filamentous fungi can also contribute to the digestion of wood by the insects^3,9^. Xylose-fermenting yeasts are associated with passalid beetles (Passalidae), stag beetles (Lucanidae), and longhorned beetles (Cerambycidae), which are all known to colonize and feed on woody tissue^10–12^. In most cases, however, the components of tissues in wood that are utilized by fungal symbionts remain poorly understood.

The lizard beetle *Doubledaya bucculenta* Lewis (Coleoptera, Erotylidae, Languriinae) is endemic to Japan and uses internodes of recently dead culms of bamboos (Poaceae) in the genera *Pleioblastus* Nakai and *Semiarundinaria* Makino as sites of oviposition^13–16^. This insect farms the Saccharomycetes yeast *Wickerhamomyces anomalus* (E.C. Hansen) Kurtzman, Robnett & Bas.-Powers on the inner surface of the internode cavities of these bamboos^17,18^. The female carries the yeast using a specialized pocket-like organ for fungal transportation (termed the mycetangium) located in the abdomen^17^. In spring, a female adult excavates a bamboo internode wall using her mandibles. After the hole reaches the internode's cavity, she inserts her ovipositor into the cavity through the hole and deposits both an egg and the symbiotic yeast on the inner surface of the fungus-free cavity^17,18^. The hatched larva spreads the yeast all over the inner wall of the internode and establishes a fungal garden where *W. anomalus* monopolizes^17,18^. Within the internode, only a single individual develops by feeding on the yeast and reaches adulthood^19^. The females incorporate the yeast of their natal gardens into their mycetangia^17^, and emerge from the internodes in spring.

As *W. anomalus* grows on the inner surface of bamboo cavities, the yeast would use carbon and nitrogen sources that are present in the inner surface tissues including pith. In culture, some *W. anomalus* strains originating from non-*D. bucculenta* assimilate xylose, arabinose, and galactose^20^, all of which are generally present in bamboo cell walls^21,22^. It is predicted that the yeast symbiont can utilize indigestible monosaccharides and polysaccharides including cellulose and hemicelluloses in the inner surface of host bamboo cavities. Alternatively, the yeast may not use these indigestible sugars but digestible sugars only. However, how the yeast assimilates these bamboo-associated carbon sources remains unknown. Moreover, it is poorly understood what carbon sources are present and how much nitrogen is contained in the inner surface of bamboo cavities.

The objective of this study was to determine what carbon sources of the host bamboo are nutritional substrates of the yeast symbiont of *D. bucculenta*. We analyzed free sugars, which are present as mono- and disaccharides, and structural sugars, which are present as polysaccharides, in pith tissues of bamboos. We also analyzed them in xylem tissues for comparison as fungal substrate. For pith, to determine what sugars are consumed by the yeast and larva, we compared sugar compositions among fresh pith, yeast-cultured pith, and larva-reared pith. To reveal the enzymatic capabilities of the yeast, we conducted carbon assimilation tests of the yeast. We additionally examined the proportions of carbon, hydrogen, and nitrogen in these bamboo tissues to determine whether bamboo-originated nitrogen is used by the yeast. Finally, the significance of yeast farming on woody substrate is discussed.

## Results

### Component analyses of bamboo tissues

To determine the difference of sugar components among internode conditions, we analyzed four different internode tissue types of *Pleioblastus simonii* bamboo: fresh pith (FP), fresh xylem (FX), pith on which the yeast symbiont of *D. bucculenta* had grown (YP), and pith on which the yeast and larvae of *D. bucculenta* had grown (LP).

Results of component analyses for total extractable sugars (i.e., free sugars), other extractives, total structural sugars derived from polysaccharides, and sulfuric acid lignin are summarized in Table 1, showing their weight ratios in the four types of powdered samples. Major components were structural sugars of polysaccharides, making up 57–64% of the weight. The values obtained in this study generally agreed with the previously reported values for several bamboo species^23^ showing that the major component ratio was not so different from those of woody plants^24,25^. The amounts of total extractable sugars were greater in either FX or FP than in YP or LP (Steel–Dwass test, *P* < 0.05) (Table 1; Supplementary Tables S1, S2). The sulfuric acid lignin content of FX was larger than that of FP and YP (Steel–Dwass test, *P* < 0.05) (Table 1; Supplementary Tables S1, S2). Total structural sugar and other extractive amounts were not significantly different among tissue types (Kruskal–Wallis test, *P* > 0.05) (Table 1; Supplementary Table S1).

**Table 1:** Weight ratios of tissue components of *Pleioblastus simonii* bamboo internodes.

	Tissue type			
	FX	FP	YP	LP
Total extractable sugars	0.032 (0.018)**a**	0.044 (0.019)**a**	0.001 (0.001)**b**	0.002 (0.001)**b**
Total structural sugars*	0.577 (0.074)	0.590 (0.093)	0.638 (0.027)	0.644 (0.046)
Sulfuric acid lignin	0.200 (0.012)**a**	0.170 (0.009)**b**	0.161 (0.010)**b**	0.174 (0.011)**ab**
Other extractives**	0.148 (0.030)	0.157 (0.017)	0.174 (0.040)	0.161 (0.032)
Total	0.957	0.961	0.974	0.981

Each value represents the mean (SD) of five internode samples. Different letters in bold indicate significant differences among tissue types by the Steel–Dwass test (*P* < 0.05) after the Kruskal–Wallis test (*P* < 0.05). No bold letters indicate no significant differences among tissue types by the Kruskal–Wallis test (*P* > 0.05) except for the total.

*FX* fresh xylem, *FP* fresh pith, *YP* pith on which *Wickerhamomyces anomalus* yeast had grown, *LP* pith on which *W. anomalus* and a larva of *Doubledaya bucculenta* had grown.

*Recalculated as dehydrated structures according to sugar analyses (see Table 2).

**Calculated by subtraction of total extractable sugars from total extractives.

**Figure 1:**
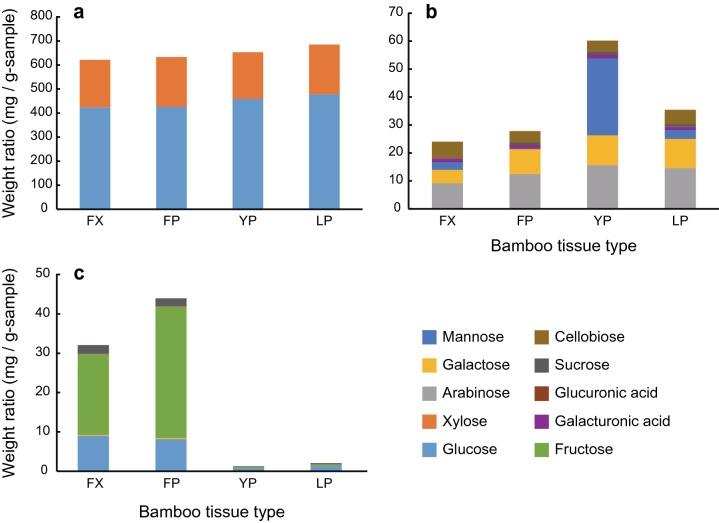
Weight ratios of structural and free sugars of *Pleioblastus simonii* bamboo internodes. (**a**) Major structural sugars (glucose and xylose). (**b**) Minor structural sugars (arabinose, galactose, mannose, galacturonic acid, glucuronic acid, sucrose, and cellobiose). (**c**) Free sugars (glucose, xylose, arabinose, galactose, mannose, fructose, galacturonic acid, and sucrose). Each column represents the mean of a given sugar of five internode samples. *FX* fresh xylem, *FP* fresh pith, *YP* pith on which *Wickerhamomyces anomalus* yeast had grown, *LP* pith on which *W. anomalus* and a larva of *Doubledaya bucculenta* had grown.

Sugar compositions (in mg g^−1^-sample) were evaluated for extractable free sugars and structural sugars hydrolyzed by sulfuric acid treatments (see summary in Table 2). Structural sugar analyses revealed that the major sugars for FX, FP, YP, and LP were glucose and xylose (Table 2; Fig. 1). As the minor ones, galactose, mannose, arabinose, galacturonic acid, glucuronic acid, sucrose, and cellobiose were detected (Table 2; Fig. 1). Cellobiose contents were quite smaller than that of glucose, indicating that an appropriate hydrolysis was conducted.

**Table 2:** Structural and free sugars of *Pleioblastus simonii* bamboo internodes.

	Tissue type (structural sugars)				Tissue type (free sugars)			
	FX	FP	YP	LP	FX	FP	YP	LP
Glucose	423.233 (48.695)	425.492 (62.624)	458.966 (48.617)	477.904 (41.435)	8.968 (5.058)**a**	8.097 (5.460)**a**	0.748 (0.473)**b**	1.351 (1.189)**b**
Xylose	197.833 (32.798)	207.242 (51.753)	193.928 (36.035)	206.765 (37.342)	n.d.	0.014 (0.032)	0.006 (0.013)	0.026 (0.058)
Arabinose	9.125 (0.875)**a**	12.432 (2.159)**b**	15.607 (2.335)**b**	14.555 (3.413)**b**	0.069 (0.020)**a**	0.098 (0.025)**a**	0.003 (0.008)**b**	0.004 (0.009)**b**
Galactose	4.831 (0.375)**a**	8.909 (1.763)**b**	10.676 (1.136)**b**	10.513 (3.606)**b**	0.211 (0.062)**a**	0.240 (0.029)**a**	0.011 (0.024)**b**	0.015 (0.027)**b**
Mannose	2.760 (1.591)**ac**	0.129 (0.288)**a**	27.562 (10.268)**b**	3.135 (1.135)**c**	0.021 (0.031)	n.d.	0.065 (0.065)	0.005 (0.012)
Fructose	n.d.	n.d.	n.d.	n.d.	20.548 (12.672)**a**	33.487 (15.152)**a**	0.283 (0.212)**b**	0.438 (0.260)**b**
Galacturonic acid	1.265 (0.172)	1.287 (0.208)	1.296 (0.185)	1.093 (0.549)	0.103 (0.096)**a**	0.102 (0.099)**a**	n.d. **a**	n.d. **a**
Glucuronic acid	0.122 (0.019)	0.114 (0.054)	0.137 (0.037)	0.112 (0.065)	n.d.	n.d.	n.d.	n.d.
Sucrose	0.283 (0.098)	0.896 (1.000)	0.958 (0.443)	0.927 (0.586)	2.144 (1.603)	1.926 (2.071)	0.180 (0.077)	0.215 (0.182)
Cellobiose	5.651 (0.763)	4.048 (1.302)	3.967 (1.392)	5.122 (1.299)	n.d.	n.d.	n.d.	n.d.

Each value represents the mean (SD) mg g^−1^-sample of five internode samples. Different letters in bold indicate significant differences of a given sugar among tissue types by the Steel–Dwass test (*P* < 0.05) after the Kruskal–Wallis test (*P* < 0.05) in either structural or free sugars. No bold letters indicate no significant differences among tissue types by the Kruskal–Wallis test (*P* > 0.05). Note that n.d. was regarded as 0 in statistical analyses.

*FX* fresh xylem, *FP* fresh pith, *YP* pith on which *Wickerhamomyces anomalus* yeast had grown, *LP* pith on which *W. anomalus* and a larva of *Doubledaya bucculenta* had grown; *n.d.* not detected.

The proportions of arabinose and galactose were significantly smaller in FX than in the others (Steel–Dwass test, *P* < 0.05) (Table 2; Supplementary Tables S3, S4; Fig. 1). In the case of mannose, YP showed the greatest proportion among tissue types (*P* < 0.05), LP was greater than FP (*P* < 0.05), while FX was not significantly different from FP or LP (*P* > 0.05) (Table 2; Supplementary Tables S3, S4; Fig. 1). The proportions of glucose, xylose, galacturonic acid, glucuronic acid, sucrose, and cellobiose were not significantly different among tissue types (Kruskal–Wallis test, *P* > 0.05) (Table 2; Supplementary Table S3; Fig. 1).

In the soluble part, the amounts of extractable free sugars were greater in either FX or FP than in YP or LP (Steel–Dwass test, *P* < 0.05). Free sugars detected from FX and FP were mainly glucose, fructose, and sucrose (Table 2; Fig. 1). Galactose, mannose, arabinose, galacturonic acid, glucuronic acid, and cellobiose were trace or not detected (Table 2; Fig. 1). The proportions of glucose, arabinose, galactose, fructose, and galactose were significantly smaller in YP and LP than in FX and FP (Steel–Dwass test, *P* < 0.05) (Table 2; Supplementary Tables S3, S4; Fig. 1). In the case of galacturonic acid, the proportion was not significantly different among tissue types (Steel–Dwass test, *P* > 0.05) (Table 2; Supplementary Tables S3, S4; Fig. 1). The proportions of xylose, mannose, and sucrose were not significantly different among tissue types (Kruskal–Wallis test, *P* > 0.05) (Table 2; Supplementary Table S3; Fig. 1).

Elemental analysis suggested that the compositions of carbon, hydrogen, and nitrogen in weight-% were not significantly different among tissue types (Kruskal–Wallis test, *P* > 0.05) (Table 3; Supplementary Table S5).

**Table 3:** The proportions of carbon (C), hydrogen (H), and nitrogen (N) and C/N ratio of *Pleioblastus simonii* bamboo tissues.

	Tissue type			
	FX (*n* = 5)	FP (*n* = 5)	YP (*n* = 5)	LP (*n* = 4)
C	46.34 (0.45)	46.28 (0.44)	46.06 (0.27)	46.42 (0.45)
H	5.57 (0.14)	5.71 (0.12)	5.73 (0.25)	5.65 (0.28)
N	0.02 (0.03)	0.13 (0.16)	0.21 (0.16)	0.07 (0.10)
C/N ratio	2106	351	217	701

Each value of C, H, and N represents the mean (SD) (%) of four or five internode samples. C/N ratio was calculated as (mean C/mean N).

*FX* fresh xylem, *FP* fresh pith, *YP* pith on which *Wickerhamomyces anomalus* yeast had grown, *LP* pith on which *W. anomalus* and a larva of *Doubledaya bucculenta* had grown.

### Carbon assimilation test

To determine the assimilation of bamboo-containing carbon sources by *W. anomalus*, 14 different carbon sources including a treatment with no carbon source were tested using liquid media. When the yeast was cultured with no carbon source for 7 days, the pellet of yeast cells was absent and the turbidity increase was 0.03. *W. anomalus* assimilated glucose, mannose, fructose, and sucrose strongly, xylose and cellobiose moderately, and corn-xylan weakly (Table 4). Its ability to assimilate corn-starch was unclear but very weak or negative (Table 4). Galactose, arabinose, galacturonic acid, glucuronic acid, and carboxymethyl cellulose were not assimilated (Table 4).

**Table 4:** Growth of *Wickerhamomyces anomalus* associated with *Doubledaya bucculenta* on different carbon sources.

Carbon source	ΔOD_600_^a^	Growth
d-Glucose	**0.50**	++
d-Galactose	− 0.03	−
d-Mannose	**0.50**	++
d-Xylose	**0.11**	+
l-Arabinose	− 0.02	−
d-Fructose	**0.41**	++
d-Galacturonic acid	− 0.01	−
d-Glucuronic acid	− 0.03	−
Sucrose	**0.53**	++
Cellobiose	**0.23**	+
Starch (corn)	0.04	w/−
Xylan (corn)	**0.08**	w
Carboxymethyl cellulose	0.00	−

Data in bold indicate that pellets of yeasts could be observed.

− no growth, *w* weak growth, + moderate growth, ++ strong growth.

^a^Difference of the turbidity increase between culture media containing no and a given carbon source.

## Discussion

Most free sugars of pith (FP) such as glucose, galactose, arabinose, and fructose decreased after *W. anomalus* (YP) and *W. anomalus* and the larva (LP) had grown, while no significant difference was found between YP and LP. This suggests that these free sugars were consumed by the yeast. FP contained glucose and fructose abundantly among the free sugars detected, and they decreased markedly in YP and LP. Carbon assimilation tests showed that *W. anomalus* assimilated glucose and fructose strongly in culture. These results suggest that *W. anomalus* utilizes mainly glucose and fructose as nutritional substrates among free sugars in bamboo internodes.

In contrast, minor free sugars such as galactose and arabinose were not assimilated in culture. This result was not supported by component analysis. This incongruence may be due to different durations of incubation of the yeast. We incubated the yeast for 47 to 73 days for the component analysis, but only for 7 days for the assimilation test. However, it may be possible that the short-term incubation did not detect the delayed assimilation ability of *W. anomalus*.

In the case of structural sugars of pith samples, in contrast, no clear difference was observed among tissue types except for mannose. FP contained little mannose but YP had large amounts of it, and LP did in small amounts. Generally, mannose is one of the major component sugars of the yeast cell wall^26^. Larvae of *D. bucculenta* obligately depend on *W. anomalus* for food^17^. Therefore, it is suggested that mannose was biosynthesized from the pith of bamboo by *W. anomalus*, and that the larvae consumed *W. anomalus* containing rich mannose. In culture, *W. anomalus* assimilated glucose and sucrose strongly, xylose and cellobiose moderately, and corn-xylan weakly, suggesting that *W. anomalus* may utilize bamboo-associated mono-, di- and polysaccharides. In the structural sugar analyses, however, no sugars decreased in YP and LP compared with FP. These contrasting results suggest that *W. anomalus* may not use these structural sugars but free sugars on bamboos in the mutualism. Alternatively, *W. anomalus* may use these sugars distributed on the top surface of pith shallowly. Generally, yeasts are unicellular organisms and thus it is very likely that they are only colonizing the surface of the examined samples, and are, in contrast to filamentous fungi, not able to grow inside the sample to gather nutrients from here. It is possible that the pith tissues used for the chemical analyses in this study were much thicker than the yeast-utilizing layer, and thus the effect of the structural sugar consumption by the yeast was masked. Moreover, the methods for preparing YP and LP should be considered. In nature, the reproduction period of *D. bucculenta* is from April to May, and eclosion occurs from late August to early October (WT, personal observation). Some larvae overwinter and pupate in the following year^17^. In the present study, however, the duration of incubation of YP was 47 days and that of LP was 47 and 73 days. Therefore, such short incubation of YP and LP may have resulted in little consumption of the structural sugars by the yeast.

When comparing the composition of sugars between pith and xylem, the proportions of free sugars detected were not significantly different. Those of some minor structural sugars were different (arabinose and galactose, pith > xylem). *W. anomalus* was incapable of assimilating arabinose and galactose. Thus, in the context of sugar contents, pith and xylem are similar substrates for the yeast. However, xylem contained more lignin than pith. The effect of lignin on the growth of *W. anomalus* should be examined in the future to determine the quality of pith and xylem as yeast substrates.

Generally, the protein weight ratio can be evaluated by nitrogen (%) with a proportional factor (e.g., 6.25)^27^. As shown in Table 3, the amount of nitrogen was the greatest in YP (mean) but the difference was not significant among tissue types. We expected a nitrogen decrease from FP or YP to LP by the larva growing. A difference was noted in the mean values, but it was not significant.

With the proportional factor 6.25, the protein weight-ratio in YP is ca. 1.3% and should be included in the sulfuric acid lignin fraction in Table 1. This value is small if the sample was mainly yeast, which is expected to contain proteins over 40% (dry weight)^28^. Therefore, the yeast ratio of the pith samples should be low and this might be one of the reasons for this unclear result. In order to discuss the nitrogen cycle in more detail, technical improvements in experimental methods, such as sampling only near the extreme surface of the pith as a thin section, will be necessary.

This study revealed that the yeast symbiont of *D. bucculenta* proliferates using mostly free sugars, mainly glucose and fructose, present in the pith of host bamboo internodes. The amount of these free sugars in the pith may affect the growth of *D. bucculenta* larvae through the yeast growth. Wood is nutrient-poor and an indigestible polymer-rich resource^4^, and thus it has been suggested that symbiotic microbes that can utilize wood aid larvae of a number of wood-inhabiting insects in the digestion of wood^5,6,9^. Fungal symbionts of some ambrosia beetles are capable of decomposing indigestible polymers such as cellulose, hemicelluloses, and lignin in woody tissue^3^. Similarly, fungal symbionts of leaf-cutter ants and fungus-farming termites that use plant materials as nutritional substrates for their mutualistic fungi can utilize these indigestible polymers, leading to the degradation of plant materials in their fungal gardens^3^. In *D. bucculenta*-yeast mutualism in the bamboo internode cavity, however, the yeast may not use bamboo-associated indigestible sugars but digestible free sugars, although the yeast has the ability to assimilate bamboo-associated indigestible sugars including xylan and xylose. The yeast may not contribute to the degradation of bamboo-associated polysaccharides through its mutualistic association with *D. bucculenta*. The yeast is commonly found in various environments in nature^20^, and as such, it may have a non-exclusive relationship with *D. bucculenta*, refraining from developing physiological interactions that provide a clear benefit for both members of the association. Our findings suggest that a symbiont’s potential abilities (e.g., xylose- and xylan-assimilating) may not always benefit its host (e.g., larval growth) in nature.

## Materials and methods

### Insects and bamboos

Five internodes (length: mean ± SD = 44.8 ± 1.1 cm, *n* = 5; diameter in the middle part of internodes: 21.4 ± 0.8 mm, *n* = 5) of five living mature culms of *P. simonii* bamboo were sampled at Kawaminami, Miyazaki Prefecture, Japan [32°9′ N, 131°29′ E] on 6 June, 2019. Per internode, four semi-cylindrical strips (ca. 15 × 2 cm) were made and stored at − 25 °C until use.

To obtain fungus-free larvae of *D. bucculenta*, we sampled five beetle eggs from *P. simonii* bamboo obtained at Toyota, Aichi Prefecture, Japan [35°9′ N, 137°13′ E] on 9 May, 2019 in the laboratory from ovipositing females collected at Kawaminami on 10 and 11 April, 2019. The eggs were immersed in 99.5% ethanol for 10 s followed by 70% ethanol for 10 s for surface sterilization and then individually placed on potato dextrose agar (PDA) (Difco, Detroit, MI, USA) plates. The plates were incubated at 25 °C in the dark until 30 days after larval hatching to confirm the absence of the formation of yeast or other microbial colonies. Consequently, all five larvae hatched successfully and aseptically.

The bamboo used in this study was morphologically identified using the literature^29^. This is native to the study areas and no other host bamboo species are distributed there^29^. Therefore, no voucher specimen of this bamboo has been deposited in a publicly available herbarium. No specific permits were required for the described field studies. The location is not privately-owned or protected in any way. The field studies did not involve endangered or protected species. All applicable international, national, and/or institutional guidelines for the care and use of animals and plants were followed. This study is reported in accordance with ARRIVE guidelines.

### Component analyses of bamboo tissues

For YP and LP, the yeast *W. anomalus* originating from *D. bucculenta* in Kawaminami (strain: DBL05Kawaminami) was cultured on a 9-cm PDA plate to obtain enough biomass for further experiments. Afterwards, yeast cells were suspended in ca. 10 mL of sterilized water, and were inoculated on the inner surface of the autoclaved internode strips using an autoclaved tissue paper immersed with the yeast suspension. For LP, additionally, the fungus-free 2nd instar larvae (weight: mean ± SD = 2.4 ± 0.4 mg, *n* = 5) were individually placed on the yeast-inoculated strips. Each of these yeast-inoculated and yeast-and-larva-inoculated strips was then put in a sterilized test tube (3.0 cm in diameter and 20 cm tall) with moistened cotton placed at the bottom. Each of the test tubes was covered with a sterilized polypropylene cap, sealed with Parafilm Sealing Film (Pechiney Plastic Packaging, Chicago, IL, USA) on which three small holes were made using a fire-sterilized insect pin to avoid oxygen shortage, and individually put in a plastic zipper bag. These yeasts and insects were incubated at 25 °C in the dark for 47 days for YP (*n* = 5), and 47 (*n* = 4) and 73 (*n* = 1) days until these larvae reached adulthood for LP (adult elytral length: mean ± SD = 9.2 ± 0.4 mm, *n* = 5). Microbial contamination was invisible to the naked eye.

For FP, YP and LP, the inner surface (up to 0.3 mm in thickness, dry weight: 336 to 935 mg) of a strip was sampled using a small U-shaped gouge. In the case of FX, first, the pith of a strip was completely removed, and then xylem tissue (up to 0.5 mm in thickness, dry weight: 729 to 872 mg) was sampled using a small U-shaped gouge. These tissues were individually sampled from five strips derived from five different internodes for each tissue type.

Samples were extracted by aqueous ethanol and hydrolyzed by sulfuric acid with reference to the literature^30–32^ as follows. Four types of samples were freeze-dried and pulverized using a rotor-speed mill (Fritsch, PULVERISETTE 14, 0.2 mm mesh). About 80 mg of powdered sample was extracted using 5-mL 80% ethanol aqueous solution (aq.) at 63 °C three times. The volume of the extracts was adjusted to 25 mL, filtered, and analyzed using ion exchange chromatography measurements (extractable sugar analysis). Their extracted residues were hydrolyzed using sulfuric acid as follows: 50-mg samples were immersed in 1.64-g 72% sulfuric acid aq. at 30 °C for 2 h, boiled in 39.4-g 3% sulfuric acid aq. for 4 h, and filtered to collect sulfuric acid residues as sulfuric acid lignin fractions. The volumes of the filtrates were fixed to 100 mL, passed through a sulfuric acid-removing filter (DIONEX OnGuard IIA), and submitted to ion exchange chromatography measurements (structural sugar analysis). For the uronic acid measurements, the sulfuric acid-removing filter was not used.

Ion exchange chromatography measurements were conducted using a DIONEX ICS-3000 apparatus. The measurement conditions were as follows: column, CarboPac PA-1 (2.0 mm I.D. × 250 mm L, Dionex corp.); flow rate, 0.3 mL min^−1^; column temperature, 30 °C; injection volume, 25 µL; eluent, H_2_O (solvent A), 100 mM NaOHaq. (solvent B), aqueous solution containing 100 mM NaOH and 1.0 M CH_3_COONa (solvent C), and aqueous solution containing 100 mM NaOH and 150 mM CH_3_COONa (solvent D). The gradient conditions for monomers, dimers, and uronic acids were as follows: for monomers, with a gradient of B 0.5% C 0% 45 min, C 100% 10 min, B 100% 10 min, B 0.5% C 0% 20 min; for dimers, with a gradient of B 50% C 0% 50 min, C 100% 10 min, B 100% 10 min, B 50% C 0% 15 min; for uronic acids, with a gradient of D 100% 10 min. These extraction, hydrolysis, and measurement procedures were conducted using *n* = 5 samples. For the structural sugars, their yield was calculated as the dehydrated state. The values of other extractives % were calculated by the subtraction of total extractable sugars % from total extractives %.

Elemental analysis (carbon, hydrogen, nitrogen) was conducted by 2400 CHNS Organic Elemental Analyzer (PerkinElmer Japan, Yokohama, Japan). About 1-mg dried samples were burned completely and the produced CO_2_, H_2_O, and N_2_ (after reduction of NOx species) gasses were quantified by a thermal conductivity detector.

Means of components of bamboo tissues were compared among tissue types using the Steel–Dwass test after the Kruskal–Wallis test. Calculations were performed using R 3.5.1^33^.

### Carbon assimilation test

The yeast *W. anomalus* (DBL05Kawaminami) was cultured aerobically in 20 mL of yeast nitrogen base (YNB) (Difco) containing 0.5% glucose at 25 °C in the dark for 2 days with shaking at 85 rpm. The culture media were centrifuged and cell pellets were suspended in sterile water, in which the OD_600_ was adjusted to 0.10. Fifty μL of the cell suspension was added into a tube (2 mL) with 1 mL of each of 14 different media containing YNB and one of the following carbon sources: d-glucose, d-galactose, d-mannose, d-xylose, l-arabinose, d-fructose, d-galacturonic acid, d-glucuronic acid, sucrose, cellobiose, starch from corn, xylan from corn, carboxymethyl cellulose, and no carbon source (*n* = 5 to 6). The concentration of each carbon source was 0.5 g L^−1^, except for xylan at 1.5 g L^−1^. The tubes were shaken at 85 rpm and incubated at 25 °C in the dark for 7 days. Afterwards, the presence of visible pellets of yeasts and OD_600_ were recorded to determine the growth of the strain. The degree of assimilation was scored according to the presence of the pellets and the difference in the turbidity increase (ΔOD_600_) between culture media containing no and a given carbon source as follows: no growth (without a pellet, ΔOD_600_ < 0.03), weak growth (with a pellet, 0.03 ≤ ΔOD_600_ < 0.10), moderate growth (with a pellet, 0.10 ≤ ΔOD_600_ < 0.40), and strong growth (with a pellet, 0.40 ≤ ΔOD_600_ < 1.00)^8^.

## Supplementary Information

Supplementary Information.

## Data Availability

All datasets in the current study are available from the corresponding author on reasonable request.
