# Supplementary material for: Nutritional resources of the yeast symbiont cultivated by the lizard beetle Doubledaya bucculenta in bamboos
Source: Sci Rep. 2021 Sep 28;11:19208. doi: 10.1038/s41598-021-98733-y (PMC8479059; doi:10.1038/s41598-021-98733-y)
Supplement: Supplementary file 1 — Supplementary Information. [file 41598_2021_98733_MOESM1_ESM.pdf]

Supplementary information

Nutritional resources of the yeast symbiont cultivated by the lizard beetle *Doubledaya bucculenta* in bamboos

Wataru Toki and Dan Aoki

Supplementary Table S1. Comparisons of weight ratios of tissue components of *Pleioblastus simonii* bamboo among tissue types by the Kruskal–Wallis test.

	χ^2	d.f.	*P*-value
Total extractable sugars	15.114	3	**0.002**
Total structural sugars	5.606	3	0.133
Sulfuric acid lignin	14.040	3	**0.003**
Other extractives	2.246	3	0.523

Significant differences ($P < 0.05$) in weight ratios among types are highlighted in bold.

Supplementary Table S2. Pairwise comparisons of weight ratios of tissue components of *Pleioblastus simonii* bamboo between tissue types by the Steel–Dwass test.

	FX vs. FP	FX vs. YP	FX vs. LP	FP vs. YP	FP vs. LP	YP vs. LP
Total extractable sugars	0.526	**0.045**	**0.045**	**0.045**	**0.045**	0.659
Sulfuric acid lignin	**0.045**	**0.045**	0.077	0.194	0.783	0.125

Significant differences ($P < 0.05$) in weight ratios between types are highlighted in bold. *FX* fresh xylem, *FP* fresh pith, *YP* pith on which *Wickerhamomyces anomalus* yeast had grown, *LP* pith on which *W. anomalus* and a larva of *Doubledaya bucculenta* had grown.

Supplementary Table S3. Comparisons of weight ratios of structural and free sugars of *Pleioblastus simonii* bamboo among tissue types by the Kruskal–Wallis test.

	χ^2	d.f.	*P*-value
Structural sugars			
Glucose	2.794	3	0.424
Xylose	0.429	3	0.934
Arabinose	12.806	3	**0.005**
Galactose	12.131	3	**0.007**
Mannose	15.101	3	**0.002**
Galacturonic acid	0.417	3	0.937
Glucuronic acid	1.389	3	0.708
Sucrose	6.200	3	0.102
Cellobiose	4.966	3	0.174
Free sugars			
Glucose	14.429	3	**0.002**
Xylose	1.139	3	0.768
Arabinose	16.133	3	**0.001**
Galactose	15.134	3	**0.001**
Mannose	5.824	3	0.121
Fructose	15.800	3	**0.001**
Galacturonic acid	7.822	3	**0.050**
Sucrose	5.217	3	0.157

Significant differences ($P < 0.05$) in weight ratios among types are highlighted in bold.

Supplementary Table S4. Pairwise comparisons of weight ratios of structural and free sugars of *Pleioblastus simonii* bamboo between tissue types by the Steel–Dwass test.

	FX vs. FP	FX vs. YP	FX vs. LP	FP vs. YP	FP vs. LP	YP vs. LP
Structural sugars						
Arabinose	**0.045**	**0.045**	**0.045**	0.194	0.783	0.885
Galactose	**0.045**	**0.045**	**0.045**	0.285	0.885	0.989
Mannose	0.148	**0.045**	0.954	**0.036**	**0.036**	**0.045**
Free sugars						
Glucose	1.000	**0.045**	**0.045**	**0.045**	**0.045**	0.885
Arabinose	0.285	**0.036**	**0.036**	**0.036**	**0.036**	0.999
Galactose	0.885	**0.036**	**0.041**	**0.036**	**0.041**	0.918
Fructose	0.194	**0.045**	**0.045**	**0.045**	**0.045**	0.526
Galacturonic acid	1.000	0.216	0.216	0.216	0.216	N.A.

Significant differences ($P < 0.05$) in weight ratios between types are highlighted in bold. *FX* fresh xylem, *FP* fresh pith, *YP* pith on which *Wickerhamomyces anomalus* yeast had grown, *LP* pith on which *W. anomalus* and a larva of *Doubledaya bucculenta* had grown, *N.A.* not applicable.

Supplementary Table S5. Comparisons of proportions of carbon (C), hydrogen (H), and nitrogen (N) of *Pleioblastus simonii* bamboo tissues among tissue types by the Kruskal–Wallis test.

	χ^2	d.f.	*P*-value
C	2.160	3	0.540
H	1.495	3	0.683
N	4.491	3	0.213
